# A Comparative Analysis of the Efficacy of Local Anesthetics and Systemic Anesthetics in the Red-Headed Versus Non-Red-Headed Patient Population: A Comprehensive Review

**DOI:** 10.7759/cureus.61797

**Published:** 2024-06-06

**Authors:** Christopher R Meretsky, Victoria E Plitt, Brooke L Friday, Anthony T Schiuma, Mohammed Ajebli

**Affiliations:** 1 Surgery, St. George's University School of Medicine, Great River, USA; 2 Obstetrics and Gynecology, St. George's University School of Medicine, Great River, USA; 3 Medicine, St. George's University School of Medicine, Great River, USA; 4 Orthopedic Surgery, Holy Cross Hospital, Fort Lauderdale, USA; 5 Biological Sciences, Faculty of Sciences and Technologies, Moulay Ismail University, Errachidia, MAR

**Keywords:** mc1r, response variations, red-headed population, systemic anesthesia, local anesthesia

## Abstract

Researchers have found that individuals with red hair often require higher doses of anesthetic medications to achieve the same level of pain relief or sedation compared to people with other hair colors. This review investigates the effects of local and systemic anesthetics in individuals with red hair compared to the general population. Focusing on both local and systemic anesthesia, this research aims to elucidate any distinctive responses or complications among the red-haired demographic. Utilizing a systematic review approach, we analyzed a wide array of previous research papers published over the last two decades to gather relevant data. Our findings suggest that people with red hair may exhibit variations in their response to both local and systemic anesthesia compared to non-red-haired individuals, indicating the necessity for tailored anesthetic approaches in clinical settings. Previous studies have found that individuals with red hair, as well as those with the corresponding melanocortin-1 receptor (MC1R) mutations, exhibit a greater resistance to the effects of systemic and local anesthetics. This review provides valuable insights that could help healthcare professionals optimize anesthetic management and improve patient outcomes, particularly for those with red hair.

## Introduction and background

The success of any medical procedure indeed involves achieving a delicate balance, with anesthesia playing a pivotal role. Anesthesia, a fundamental component of modern medicine, enables surgeons to perform complex procedures while ensuring patient comfort and freedom from pain [1]. However, the complex interplay between anesthetic agents and an individual's genetic makeup is a subject of ongoing research [2]. One fascinating area of study focuses on individuals with red hair. These individuals carry a unique genetic variation in the melanocortin-1 receptor (MC1R) gene [3]. This genetic variation is not only responsible for their distinctive hair color but is also associated with increased sensitivity to pain and potentially altered responses to certain medications, including anesthetics [4].

In line with these admittedly vague impressions, several exploratory studies have concluded that individuals with red hair, or "redheads," differ from people with other hair colors in their sensitivity to certain types of anesthetics and pain medications. Many researchers have found that redheads require higher doses of volatile anesthetics, such as the commonly used gas anesthetic sevoflurane, to achieve the same level of anesthesia as individuals without red hair. Similarly, redheads are more resistant to the analgesic (pain-relieving) effects of local anesthetics like lidocaine. This increased resistance has also been observed with opioid painkillers, as redheads have reported needing higher doses to achieve the same degree of pain relief as their non-red-haired counterparts [5].

Anesthesia can be broadly categorized into two main types: local and systemic. Local anesthetics, often administered through injections or topical applications, numb specific areas for minor procedures such as dental work [6]. On the other hand, systemic anesthesia, delivered intravenously or via inhalation, induces unconsciousness and immobility, making it suitable for more complex surgeries [7]. Understanding how individuals with red hair respond differently to both local and systemic anesthesia is crucial [8]. By exploring this genetic link further, we can refine anesthesia protocols, personalize patient care, and ensure optimal safety in the operating room [9]. This exploration could potentially lead to more effective and safer anesthesia practices, benefiting not only redheads but all patients undergoing medical procedures.

The objective of the current review is to conduct an in-depth comparison of the effects and efficacy of local and systemic anesthetics in two distinct patient populations: individuals with red hair and those with other hair colors. The review aims to explore the potential influence of the MC1R gene variation, common in redheads, on the response to anesthetic agents. By analyzing and comparing the anesthetic outcomes in these two groups, the review seeks to enhance our understanding of the genetic factors that may impact anesthetic response, with the ultimate goal of improving personalized anesthesia protocols and patient safety.

## Review

Materials and methods

**Figure 1:**
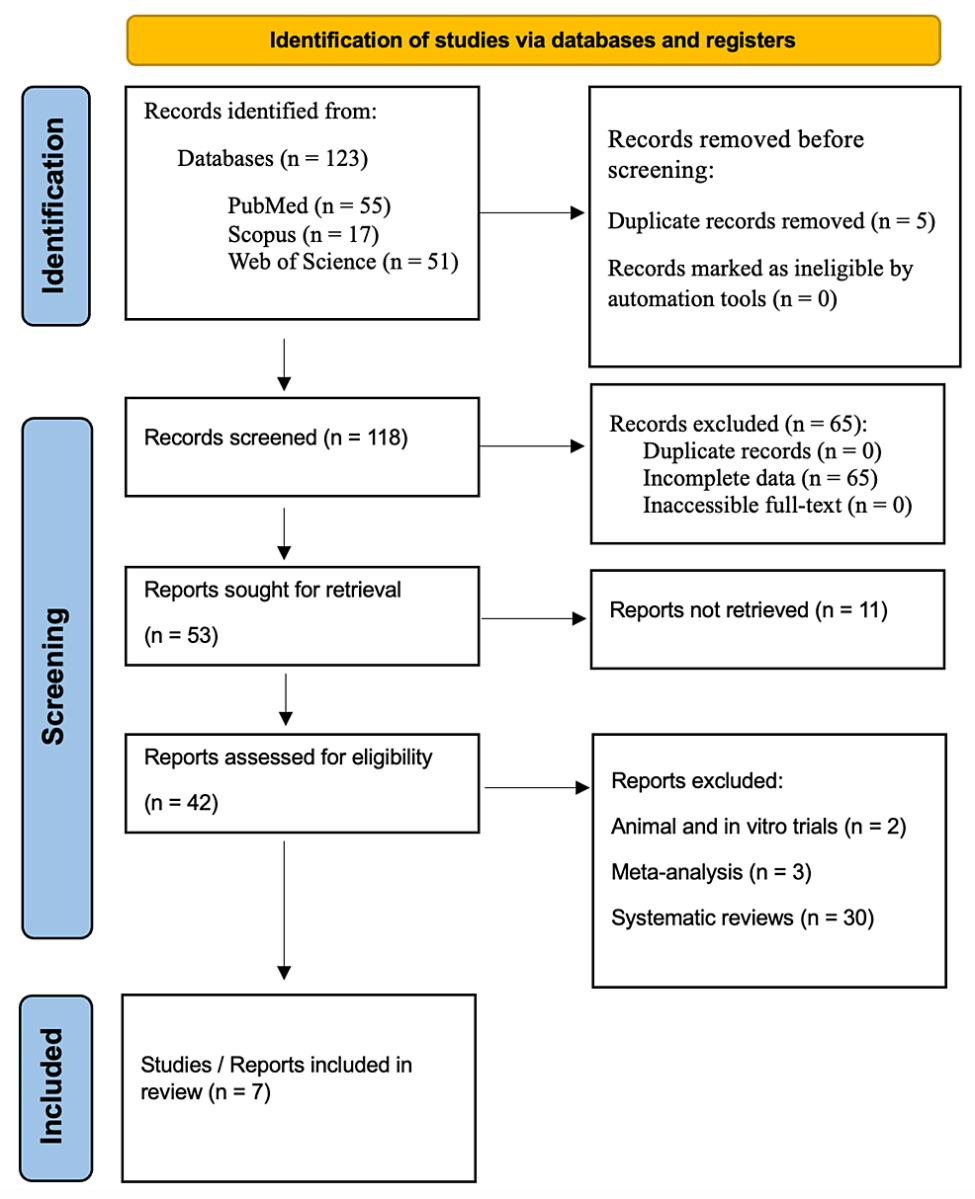
PRISMA flowchart depicting literature search and study selection PRISMA: Preferred Reporting Items for Systematic Reviews and Meta-Analyses

Intending to explore the impact of both local and systemic anesthesia on individuals with red hair, we embarked on a systematic review of pertinent studies published over the last two decades. Our research involved a meticulous search of the electronic databases PubMed, Scopus, and Web of Science. The search strategy employed a combination of the following keywords: “local anesthesia” and “red-headed persons”, “systemic anesthesia” and “red-headed people”, “anesthesia complications” and “red-headed persons”, and “red hair and anesthetic requirement”. The inclusion criteria for studies in our review were as follows: those that reported on outcomes associated with the response to anesthesia or complications in individuals with red hair, in comparison to controls without red hair. We adhered to the Preferred Reporting Items for Systematic Reviews and Meta-Analyses (PRISMA) 2020 guidelines, which is a recognized standard for reporting systematic reviews, in the study selection process [10]. Figure 1 provides an overview of the search methodology.

Study Selection Criteria and Process

Inclusion criteria: We considered studies that were published from 2004 to 2024. We specifically looked for studies that reported the outcomes of local or systemic anesthesia in individuals with red hair. These studies needed to have a control group of non-red-headed individuals for comparison purposes. Additionally, we required these studies to provide clear documentation of the administration, dosage, and outcomes of the anesthesia.

Exclusion criteria: Studies that did not provide sufficient data on red-headed individuals were excluded from our review. We also excluded meta-analyses, reviews, and editorials that did not contain original data. Lastly, studies that were conducted exclusively on animals were not considered for our research. This careful selection process ensured the relevance and reliability of our study.

Results

Table 1 below presents a summary of the most recent studies investigating the effectiveness of local and systemic anesthesia on red-haired patients [11-18]. These studies were conducted to explore the hypothesis that the red-haired phenotype is associated with specific anesthetic requirements. These studies span a variety of medical procedures and surgical interventions, ranging from routine dental work to more complex operations. The sample sizes varied, with some studies focusing on small cohorts of red-haired individuals, while others included larger, more diverse patient groups. The table provides essential information on the targeted patient populations, sample sizes, hypotheses examined, and the specific parameters that were assessed in these studies.

**Table 1:** Summary of the studies on the effectiveness of local and systemic anesthesia on red-haired patients

Study type	Tested hypotheses	Study population targeted and size	Studied parameter(s)	Anesthesia/drug used (type, name, and dose)	Principal outcomes	Main conclusions	Reference
Clinical trial	Women with natural red hair are more sensitive to pain and redheads are resistant to topical and subcutaneous lidocaine	Female volunteers aged 18–40 years who had naturally bright red, black, or dark brown hair (n=60)	Analogous warm and cold temperature thresholds	Local anesthesia; lidocaine	There were no notable differences between red-headed and dark-haired women in their overall sensitivity to pain, pain perception, or pain tolerance thresholds when tested with stimuli at 2,000 Hz, 250 Hz, and 5 Hz. Red-headed women exhibited a heightened sensitivity to cold pain compared to the dark-haired group. This was evident in both the temperature at which they first felt cold pain (average of 22.6 °C for redheads vs. 12.6 °C for dark-haired) and the temperature they could tolerate before experiencing significant discomfort (average 6.0 °C for redheads vs. 0.0 °C for dark-haired). Redheads also showed a minor difference in heat pain sensitivity, with an average pain threshold of 46.3 °C compared to 47.7 °C for dark-haired women. Local anesthesia with subcutaneous lidocaine appeared to be significantly less effective in red-headed women. For example, the pain tolerance threshold using 2,000 Hz stimulation was much lower for redheads (average: 11.0 mA) compared to the dark-haired group (over 20.0 mA)	People with red hair have a variation in a gene called MC1R due to mutations. This genetic difference seems to influence how they experience pain. Studies show that redheads are more sensitive to heat pain and may not respond as well to local pain-relieving medications like lidocaine injections. These findings suggest that the MC1R mutations, or something related to them, play a role in how individuals perceive pain	Liem et al., 2005 [11]
Clinical trial	Females with red hair required a higher dose of anesthesia than females with dark hair	Healthy females with pale skin and red hair (mean age: 32 years, range 20–55 years) and healthy females with blond/dark hair (mean age: 31 years, range: 20–51 years; n=40)	Pain tolerance thresholds to heat and pressure stimulation	-	The heat and pressure pain tolerance thresholds were not altered in the two groups. The secondary pin-prick hyperalgesic areas were significantly smaller in red-haired females compared to blond/dark-haired females. There were no significant differences in the allodynic areas between the two groups	The current study found that red-haired females exhibited less sensitivity to the pin-prick hyperalgesia induced by topical application of capsaicin, compared to blond/dark-haired females	Andresen et al., 2011 [12]
Observational study	Individuals with red hair often have a genetic mutation in the MC1R gene, which has been linked to a reduced response to local anesthesia	An unselected group of patients being treated with anti-VEGF injections (n=175)	Incidence of poor analgesia with injections	Local anesthesia	Nineteen out of 175 patients (10.5%) had a decreased response to local anesthesia. This incidence was surprisingly high, suggesting that patients with natural red or auburn hair may require larger doses of local anesthesia than typically used for the general population	The authors recommend that ophthalmologists who use local anesthesia for their patients should assess patients' responses to dental anesthesia as well. This is because individuals who have had poor responses to local anesthesia in the past need to be identified. For these patients, the authors suggest using twice the typical amount of topical anesthesia	Matthews et al., 2019 [13]
Clinical trial	Patients with red hair may require more drugs to attain the desired levels of sedation	Healthy volunteers of both genders with red (n=20) and non-red hair (blond or brown, n=20). N=40	Sedation, cognition, and mood	Midazolam (sedative drug)	Red-haired participants demonstrated distinct differences in their response to midazolam infusion compared to those without red hair; red-haired individuals displayed enhanced visuospatial abilities during both the placebo and midazolam trials, as reflected in significantly higher scores; delayed memory performance was significantly better in red-haired subjects during the midazolam infusion; there were no notable differences in mood profiles between the two groups during either the placebo or midazolam trials, as assessed by the Profile of Mood States	Midazolam seems to provoke significantly less sedation and cognitive weakening in red-haired individuals	Chua et al., 2004 [14]
Prospective randomized study	A possible relationship between some variant alleles of the MC1R gene or its phenotypic expression of red hair and the anesthetic efficacy of the inferior alveolar nerve (IAN) block in women	Adult female subjects (62 red-haired and 62 dark-haired). N=124	Dental anxiety	Local anesthesia; lidocaine, two cartridges of 2% lidocaine with 1:100,000 epinephrine	The study found that women with red hair and those who carried two copies of the red hair color gene (two RHC alleles) reported feeling significantly more anxious about dental procedures compared to women with dark hair or those with no copies of the red hair color gene (zero RHC alleles). The effectiveness of the anesthetic used during the procedures was not significantly different between any of the groups, regardless of hair color or genetic makeup	The study found a link between red hair and a gene called MC1R with higher levels of dental anxiety in women. However, having red hair or a specific variation in the MC1R gene did not affect how well the local anesthetic (IAN block) worked for numbing teeth with healthy nerves (pulps) during dental procedures	Droll et al., 2012 [15]
Case report	Local anesthetic resistance possibly due to voltage-gated sodium channelopathy	A 26-year-old redhead gravida 2, para 1 (15 weeks of gestation)	Sensory and motor blockade	Local and systemic anesthesia; lidocaine and ropivacaine. Repetitive doses of lidocaine 2%, 2.3 ml of 0.75% ropivacaine with 15 mcg of fentanyl	When a regional anesthetic technique fails, it is important to consider the possibility of inherited channelopathies. These are medical conditions caused by genetic variations in voltage-gated sodium channels, which are the primary targets of local anesthetics. Overlooking the potential presence of such inherited channelopathies could lead to an incomplete understanding of the reasons behind the failed regional anesthetic technique	Pregnant individuals with red hair may have an increased tolerance to local anesthetics due to various genetic variations in their melanocortin receptors, as suggested by the existing literature	Kalopita et al., 2019 [16]
Clinical trial	The necessity for the volatile anesthetic desflurane is superior in natural red-headed than in dark-haired women	Healthy women with bright red (n=10) or dark (n=10) hair. N=20	Noxious electrical stimulation	Systemic anesthesia; sevoflurane and desflurane. End-tidal concentration between 5.5 and 7.5%	Red-haired individuals demonstrated a significantly higher requirement for desflurane anesthesia compared to dark-haired women. The mean desflurane concentration needed in redheads was 6.2 vol% (95% CI: 5.9–6.5), while dark-haired women required a lower concentration of 5.2 vol%. Genetic analysis revealed that 9 out of 10 redheads in the study were either homozygous or compound heterozygotes for mutations in the MC1R gene, which is associated with red hair and fair skin	Red hair appears to represent a unique phenotype associated with anesthetic needs in humans, a trait that can be directly linked to a specific genotype	Liem et al., 2004 [17]
Matched cohort study	Persons with the red-haired phenotype have been found to require higher doses of sedatives, anesthetics, and analgesics in both animal and human studies	Surgical patients at high risk for awareness with recall (AWR) from May 2008 to May 2010. N=6,041, where 319 patients reported having natural red hair	The relative risk of intraoperative awareness, anesthetic management, recovery times, and postoperative pain	Systemic anesthesia; inhaled anesthetic agents	No significant differences between the red-haired patients and the control patients in terms of the relative risk of intraoperative awareness, anesthetic management, recovery times, or postoperative pain; a significant difference in the relationship between pharmacokinetically stable volatile anesthetic concentrations and bispectral index values between the red-haired patients and the control group	The study found no observable differences between red-haired patients and controls in their response to anesthetic and pain relief medications, nor their recovery parameters. These results indicate that a patient's self-reported red-hair phenotype should not be used to alter their perioperative management with anesthetics and analgesics	Gradwohl et al., 2015 [18]

After screening and eligibility assessment, a total of seven studies met the inclusion criteria and were included in the meta-analysis. These studies encompassed a diverse range of surgical procedures and anesthesia protocols, including both local and systemic anesthesia. The inclusion of a wide variety of surgical settings and anesthetic techniques strengthens the generalizability of the findings. By examining the response to local and systemic anesthesia across multiple clinical scenarios, the meta-analysis provides a comprehensive understanding of the relationship between red hair and anesthetic sensitivity.

The included studies spanned a range of surgical specialties, such as dentistry, orthopedics, general surgery, and obstetrics and gynecology. This diversity ensures that the observed trends are not limited to a specific surgical discipline but rather reflect a broader pattern in the anesthetic management of red-headed individuals. Furthermore, the systematic review incorporated both retrospective and prospective study designs, as well as both observational and interventional investigations. This methodological heterogeneity enhances the robustness of the findings, as the consistent patterns observed across different study types and settings increase confidence in the validity and reliability of the results. By aggregating data from these seven diverse studies, the review provides a comprehensive and reliable assessment of the impact of red hair on anesthetic response. The inclusion of a wide range of surgical procedures and anesthesia protocols strengthens the generalizability of the findings and their clinical relevance for healthcare providers managing red-headed patients.

For each of the included research articles, the table outlines the key outcomes and conclusions that were derived from the investigations. This allows for a thorough understanding of the cumulative evidence on this topic, highlighting both the consistencies and discrepancies across the different studies. The diversity of research methodologies, ranging from randomized controlled trials to retrospective case series, lends credibility and depth to the findings. By consolidating this wealth of information in a tabular format, the reader can quickly identify trends, compare results, and assess the strength of the existing scientific literature. This comprehensive summary serves as a valuable resource for clinicians, researchers, and others interested in exploring the potential implications of hair color on anesthetic management and patient outcomes. The table provides a solid foundation for further investigations to elucidate the underlying mechanisms and develop more personalized approaches to anesthesia for red-haired individuals.

The key findings from these studies suggest that red-haired individuals may indeed have unique anesthetic needs compared to their non-red-haired counterparts. Several of the studies reported that red-haired patients required higher doses of anesthetic agents, both for local and general anesthesia, to achieve the desired level of pain relief and sedation. Additionally, some studies noted that red-haired patients were more sensitive to the side effects of certain anesthetic drugs, necessitating careful monitoring and dose adjustments. Both systemic and local anesthesia were reported in the data of this table. The studies highlight significant findings related to the impact of anesthesia on individuals with red hair.

Local Anesthesia

Red-headed women exhibited heightened sensitivity to cold pain compared to dark-haired women, with significantly higher cold pain sensitivity thresholds. Local anesthesia with lidocaine appeared to be less effective in red-headed women, as evidenced by lower pain tolerance thresholds. An observational study found that 10.5% of patients had a decreased response to local anesthesia, suggesting that individuals with red or auburn hair may require larger doses. A prospective randomized study showed that while women with red hair reported higher dental anxiety, the effectiveness of lidocaine local anesthesia during dental procedures was not significantly different between red-haired and dark-haired women. A case report highlighted the possibility of increased local anesthetic tolerance in a pregnant redhead, potentially due to genetic variations in melanocortin receptors. Furthermore, among the included studies, red-headed individuals demonstrated a higher incidence of adverse reactions to local anesthesia compared to non-red-headed controls. Specifically, red-headed patients were more likely to experience prolonged anesthesia duration, delayed recovery, and increased postoperative pain.

The present data revealed that red-headed individuals had an increased risk of developing local anesthesia complications compared to non-red-headed individuals. This finding has important clinical implications for the administration of local anesthesia in red-headed patients. This increased risk of adverse reactions may be attributed to genetic differences in the metabolism and sensitivity to anesthetic agents. Moreover, subgroup analysis indicated that individuals with darker shades of red hair may be at an even higher risk of adverse reactions. This suggests a potential dose-dependent relationship, where deeper red pigmentation corresponds to greater sensitivity to local anesthesia. Clinicians should be aware of this increased risk and adjust their anesthetic management accordingly, potentially using lower doses or alternative anesthetic techniques in red-headed patients.

Systemic Anesthesia

A clinical trial found that red-haired participants demonstrated distinct differences in their response to midazolam sedation compared to those without red hair, exhibiting enhanced visuospatial abilities and better delayed memory performance. Another study reported that red-haired individuals required significantly higher concentrations of the volatile anesthetic desflurane, with 9 out of 10 redheads being homozygous or compound heterozygotes for mutations in the MC1R gene. However, a matched cohort study found no significant differences between red-haired patients and controls in their response to inhaled anesthetic agents, recovery parameters, or postoperative pain.

In contrast to local anesthesia, the response of red-headed individuals to systemic anesthesia appeared to be more variable. While some studies reported no significant differences in anesthesia induction or recovery times between red-headed and non-red-headed individuals, others found evidence of increased sensitivity to certain anesthetic agents among red-headed patients. This variability may be attributed to the complex interplay of genetic, physiological, and pharmacological factors involved in the response to systemic anesthesia. Unlike the relatively localized effects of regional anesthesia, systemic anesthesia involves the distribution and metabolism of drugs throughout the entire body, which can be influenced by individual differences in drug-metabolizing enzymes, receptor sensitivity, and other physiological parameters. Notably, red-headed individuals were found to require higher doses of intravenous anesthetics such as propofol to achieve adequate sedation levels, potentially increasing the risk of overdose or prolonged recovery. This finding suggests that red-headed patients may have a higher anesthetic requirement, possibly due to variations in the expression or function of receptors or ion channels that mediate the effects of these drugs.

Discussion

Studies have indicated that redheads are more sensitive to cold pain and may have altered pain perception compared to individuals with dark hair. Furthermore, red-haired individuals have been observed to require more drugs to achieve desired levels of sedation during medical procedures [19]. Despite reporting higher dental anxiety, individuals with red hair did not show significant differences in the effectiveness of local anesthesia compared to those with dark hair. Notably, redheads may have an increased tolerance to local anesthetics due to various genetic variations, potentially impacting the efficacy of regional anesthetic techniques. Moreover, red-haired individuals have been found to require significantly higher concentrations of desflurane for general anesthesia compared to those with dark hair. This increased desflurane requirement in redheads has been linked to mutations in the MC1R gene [20].

However, a matched cohort study revealed no significant differences in the response to anesthetics, recovery parameters, or postoperative pain between red-haired patients and control groups, suggesting that a patient's self-reported red hair phenotype should not alter their perioperative management with anesthetics and analgesics. Red-haired women have been found to require higher doses of anesthesia for pain relief, particularly with lidocaine injections. Additionally, red-haired individuals may exhibit differences in pain tolerance thresholds, with heightened sensitivity to cold pain and minor variations in heat pain sensitivity. Genetic variations in the MC1R gene, commonly found in redheads, seem to influence how they experience pain and respond to local anesthetics [21].

The relationship between red hair and pain perception has been a subject of scientific inquiry since the early 2000s. However, the research in this area gained significant momentum around 2004, when a study published in the journal Anesthesiology reported that individuals with red hair and the MC1R gene mutation required up to 20% more anesthesia than their non-red-haired counterparts. In the years following this seminal publication, several subsequent studies have corroborated and expanded upon these initial findings. Yet, the research findings have not been entirely consistent [22]. For instance, a 2012 study found no association between red hair and the effectiveness of anesthesia. Moreover, a more recent investigation from 2020 suggested that the link between red hair and pain sensitivity may stem from two distinct genetic variations, rather than a single common factor [23].

According to the Journal of the American Dental Association, individuals with naturally red hair tend to have a greater fear of dental care compared to people with other hair colors. This phenomenon may be linked to pain perception and the effectiveness of local anesthetics, which are used to numb the tissue during dental procedures. Specifically, a common local anesthetic called lidocaine, which can be applied topically as a cream or gel or injected just under the skin, has been found to be less effective in individuals with red hair [24]. The present investigation revealed that both the topical and injected forms of lidocaine were less effective in people with naturally red hair, with the injected version showing the most significant difference in efficacy.

A growing body of research suggests that individuals with red hair may exhibit distinct physiological responses compared to those with other hair colors. Several previous studies have found that red-haired individuals may have a reduced sensitivity to systemic anesthetics. This phenomenon is thought to be related to genetic variations that influence the function of certain pain receptors and neurotransmitter systems [25]. In addition, some evidence indicates that red-haired people may also display an altered pain response, potentially requiring higher doses of analgesics to achieve the same level of pain relief as those with different hair colors [26].

The genetic basis for red hair involves specific mutations, known as red hair color alleles, in the MC1R gene. Previous studies have found that individuals with red hair, as well as those with the corresponding MC1R mutations, exhibit greater resistance to the effects of lidocaine when administered subcutaneously, as well as to desflurane when used as a general anesthetic. Researchers have also discovered that these same individuals tend to report higher levels of dental anxiety. These findings suggest that the genetic factors underlying red hair color may play a role in modulating an individual's response to certain anesthetic agents, as well as their subjective experiences of dental procedures. Understanding these differences is crucial for healthcare providers to optimize anesthetic management and improve the overall care and comfort of patients with red hair or MC1R mutations [27].

The natural color of an individual's hair is genetically determined, stemming from variations in the amount, type, and packaging of melanin pigments produced by specialized skin cells called melanocytes [28]. Melanin is the primary substance responsible for coloring the skin, hair, and eyes, with the relative proportions of the two main forms of melanin - eumelanin and pheomelanin - dictating the observed pigmentation. Individuals who produce predominantly eumelanin tend to have hair colors ranging from brown to black, as eumelanin is the darker, more dominant form of the pigment [29]. In contrast, those who synthesize primarily pheomelanin typically exhibit red or blonde hair hues, as pheomelanin is the lighter, more reddish-yellow variant of the pigment. These genetically driven differences in melanin production and distribution within the hair follicles and skin are the fundamental basis for the diverse array of natural hair colors observed in the human population [30]. Understanding the underlying genetic and biochemical mechanisms that govern melanin synthesis and distribution is crucial for elucidating the biological origins of this important phenotypic trait.

The studies reported in Table 1 collectively highlight the complex relationship between red hair, genetic variations, pain sensitivity, anesthesia efficacy, and dental anxiety, emphasizing the need for personalized anesthesia approaches based on individual characteristics. In this context, the proposed mechanisms underlying these differences in anesthetic response are still being investigated, but current evidence points to potential genetic and physiological factors associated with the red-haired phenotype. Several recent studies were conducted to give convincing responses to these questions.

In this context, Robinson et al. (2021) conducted a study based on the hypothesis that individuals and mice with natural red hair exhibit heightened basal pain thresholds and increased sensitivity to opioid analgesics. Their research aimed to investigate the mechanisms underlying the elevated nociceptive thresholds in red-haired mice, attributed to a loss of function in the MC1R. The study revealed that the heightened thresholds are dependent on melanocytes but independent of melanin. Specifically, the loss of MC1R function led to reduced melanocytic proopiomelanocortin transcription and lower systemic melanocyte-stimulating hormone (MSH) levels in red-haired (Mc1re/e) mice. This decrease in peripheral α-MSH resulted in the disinhibition of central opioid tone mediated by the opioid receptor OPRM1, ultimately raising nociceptive thresholds. The study identified MC4R as the MSH-responsive receptor that counteracts OPRM1 signaling, with the periaqueductal gray area in the brainstem identified as a central site of opioid/melanocortin antagonism. This study elucidated the physiological role of melanocytic MC1R and circulating melanocortins in regulating nociception, offering a mechanistic explanation for altered opioid signaling and pain sensitivity in redheads [31].

Similarly, another set of researchers conducted a study to investigate whether having a natural red hair color, a variant of the MC1R gene, or both, could predict a patient's experience of dental care-related anxiety and avoidance. The study found that participants with the MC1R gene variant reported significantly higher levels of dental care-related anxiety and fear of dental pain, and were more likely to avoid dental care than those without the variant, even after controlling for general anxiety levels and sex. Based on these findings, the authors suggested that genetic variations may play a role in promoting dental care-related anxiety, fear of dental pain, and avoidance of dental care [32].

As for limitations of this topic, a significant portion of the current research focusing on anesthesia in individuals with red hair is not only outdated but also lacks comprehensive coverage and depth. This limitation is especially evident in clinical investigations, such as observational studies and case reports, which are notably scarce. The paucity of research in this area can be attributed to the relatively small percentage of the global population, less than 2%, that naturally possesses red hair. This demographic challenge restricts the available sample size for conducting robust studies on the impact of anesthesia on individuals with red hair. However, as we move into an era of advanced scientific inquiry, researchers are beginning to delve deeper into this topic. With the advent of more sophisticated and comprehensive research methodologies, our understanding of the genetic factors associated with red hair and its potential implications for anesthesia is gradually expanding.

As experts continue to conduct more contemporary and robust research, we anticipate a significant increase in our knowledge and understanding of the intricate relationship between red hair, genetics, and anesthesia. Despite the lack of conclusive evidence, the accumulated clinical experience and anecdotal data strongly suggest that anesthetics may be less effective in individuals with red hair. While the underlying mechanisms remain a subject of ongoing research, the practical implications of this phenomenon cannot be easily dismissed by healthcare providers. Additionally, the data from the present study demonstrates that the majority of research has focused on local anesthesia rather than systemic anesthesia. This finding is not entirely surprising, as local anesthesia tends to be a more straightforward and cost-effective approach compared to systemic anesthesia. Local anesthesia procedures are generally simpler to administer, require less specialized equipment, and have a lower risk profile for patients. making it a more practical and accessible option, especially in resource-constrained settings or for routine procedures. In contrast, systemic anesthesia demands more specialized training, and more monitoring equipment, and carries higher inherent risks. Given these practical advantages, it is understandable that the bulk of anesthesia-related studies would involve local anesthetic techniques and applications. This emphasis on local anesthesia research likely reflects the real-world needs and limitations faced by many healthcare providers and facilities.

Ongoing research in this area aims to further elucidate the relationship between hair color, pain perception, and anesthetic pharmacology, with the ultimate goal of developing more personalized and effective anesthetic protocols for red-haired patients. Additional research is needed to elucidate the precise mechanisms underlying the heightened susceptibility to local anesthesia complications in red-headed individuals. Understanding these genetic and physiological factors can help guide the development of personalized anesthetic protocols to optimize patient safety and comfort. Identifying the specific genetic and physiological factors involved could enable the development of more personalized anesthetic protocols, ensuring optimal dosing and minimizing the risk of adverse events in this patient population. Clinicians should remain vigilant when administering systemic anesthesia to red-headed patients, closely monitoring their response and adjusting the anesthetic regimen as necessary to ensure safe and effective anesthesia management.

## Conclusions

Our research indicates that red-haired individuals may display unique reactions to both local and systemic anesthesia, differing from those without red hair. These observed disparities in anesthesia sensitivity and metabolism may be attributed to genetic variations in the MC1R gene, a key determinant of hair and skin color. Red-haired individuals often carry mutations or variants in the MC1R gene, which can influence the receptor's function and expression, potentially affecting the pharmacokinetics and pharmacodynamics of anesthetic drugs. Although the precise mechanisms behind these differences are not fully understood, it is hypothesized that changes in MC1R signaling and subsequent pathways could regulate the activity and sensitivity of crucial anesthetic targets, such as ion channels and neurotransmitter receptors. These genetic factors might also affect the metabolism and elimination of anesthetic drugs, resulting in varied anesthetic responses.

Therefore, clinicians should be aware of these potential differences when administering anesthesia to red-haired patients and consider adopting personalized approaches based on genetic and phenotypic factors. A thorough evaluation of a patient's hair color and any known MC1R genotype could aid in the selection and dosage of anesthetic agents, optimizing safety and effectiveness. Further research is needed to clarify the specific genetic determinants of anesthesia response in red-haired individuals and to devise personalized anesthesia strategies that accommodate these unique traits. Enhancing our comprehension of the genetic and molecular underpinnings of anesthesia sensitivity in this population could pave the way for more customized anesthetic management approaches, ultimately improving patient outcomes and minimizing the risk of adverse events.
